# Transient left atrial dysfunction is a feature of Takotsubo syndrome

**DOI:** 10.1186/s12968-017-0328-8

**Published:** 2017-02-06

**Authors:** Thomas Stiermaier, Tobias Graf, Christian Möller, Charlotte Eitel, Jakob Ledwoch, Steffen Desch, Matthias Gutberlet, Gerhard Schuler, Holger Thiele, Ingo Eitel

**Affiliations:** 1 University Heart Center Lübeck, Medical Clinic II (Cardiology/Angiology/Intensive Care Medicine), University Hospital Schleswig-Holstein, Ratzeburger Allee 160, 23538 Lübeck, Germany; 2 German Center for Cardiovascular Research (DZHK), Partner Site Hamburg/Kiel/Lübeck, Lübeck, Germany; 3 Department of Diagnostic and Interventional Radiology, University of Leipzig - Heart Center, Leipzig, Germany; 4 Department of Internal Medicine/Cardiology, University of Leipzig - Heart Center, Leipzig, Germany

**Keywords:** Takotsubo, Stress cardiomyopathy, Left atrial function, Cardiovascular magnetic resonance

## Abstract

**Background:**

Takotsubo syndrome (TTS) is characterized by a transient left and/or right ventricular dysfunction as a consequence of a distinctive pattern of regional wall motion abnormalities. However, a systematic evaluation of the left atrial (LA) function in patients with TTS is lacking. The aim of the present study was therefore to comprehensively assess LA performance indexes and function in patients with TTS.

**Methods:**

We compared LA function assessed by volumetric indexes derived from fractional volume changes in cardiovascular magnetic resonance (CMR) between 125 TTS patients and 125 patients with anterior ST-segment elevation myocardial infarction (STEMI). Furthermore, recovery of LA performance was evaluated in a subgroup of 20 TTS patients with follow-up CMR data.

**Results:**

Patients with TTS demonstrated a significantly lower total LA emptying fraction (EF) [44% (interquartile range (IQR) 34–53%) versus 51% (IQR 42–56%); *p* < 0.01], passive LA-EF [21% (IQR 14–30%) versus 24% (IQR 20–29%); *p* = 0.03] and active LA-EF [29% (IQR 20–38%) versus 35% (28–42%); *p* < 0.01] compared to patients with anterior STEMI. Among the 20 TTS patients with serial CMR data, the total LA-EF significantly improved from 42% (IQR 29–48%) at the acute stage to 51% (IQR 46–59%) at follow-up (*p* < 0.01). Similarly, active LA-EF (*p* < 0.01) and passive LA-EF (*p* = 0.02) improved significantly as well.

**Conclusion:**

Compared to anterior STEMI, TTS patients demonstrated a significantly decreased LA function during the acute/subacute phase of the disease. However, impairment of LA performance seems to be transient in TTS with recovery during follow-up.

## Background

The left atrium plays a key role in the cardiac cycle by modulating left ventricular (LV) filling and performance. Left atrial (LA) function is complex and comprises 3 basic aspects: (a) a reservoir function for pulmonary venous return during ventricular systole; (b) a conduit function (passive emptying) during early ventricular diastole; and (c) a booster pump function (active contraction when sinus rhythm is present) to enhance ventricular filling during late ventricular diastole [1]. Structural and functional abnormalities of the left atrium and their prognostic impact have been described in a wide range of cardiovascular diseases including atrial fibrillation, heart failure, chronic hypertension, and acute myocardial infarction [2–6].

Patients with Takotsubo syndrome (TTS) exhibit a peculiar pattern of regional hypokinesis, akinesis, or dyskinesis affecting the apical and/or midventricular or basal segments of the left ventricle [7, 8]. Furthermore, right ventricular involvement has been reported in about one-third of TTS patients [8]. These wall motion abnormalities cause a transient impairment of ventricular function which recovers completely within several weeks [7, 8]. Despite intensive research efforts the pathophysiology of the disease has still not been fully elucidated, yet. Similarly, the reason for the local distribution of abnormal contraction while sparing other surrounding segments remains largely unknown. Therefore, it cannot be ruled out that TTS directly impacts LA performance as well. Moreover, a secondary deterioration of LA function due to the severe LV contraction disturbances seems possible given the complex interplay between the left ventricle and atrium. Recently, a small study reported decreased passive LA emptying volumes in a small cohort of TTS patients [9]. However, a systematic evaluation of LA function in a large TTS population has not been performed to date.

The aim of the present study was to comprehensively assess LA function in a cohort of consecutive patients with TTS by using cardiovascular magnetic resonance (CMR), the reference standard for measurements of cardiac chamber volumes and function [10]. Moreover, the observed values were compared with a control population consisting of patients with acute anterior ST-segment elevation myocardial infarction (STEMI), a major differential diagnosis of TTS [11].

## Methods

### Study population

A total of 178 consecutive patients were diagnosed with TTS at the University of Leipzig – Heart Center between January 2005 and December 2013. Of these, CMR imaging was not performed in 53 patients due to the following reasons: cardiogenic shock/death (*n* = 19), metallic implants (*n* = 18), claustrophobia/no informed consent (*n* = 11), and organizational error (*n* = 5). The remaining 125 patients underwent CMR imaging for diagnosis confirmation and comprised the TTS population in the present study. All patients presented with acute chest pain and/or dyspnea and fulfilled the Mayo Clinic criteria: (a) transient hypokinesis, akinesis, or dyskinesis of the LV apical and/or midventricular or basal segments extending beyond a single epicardial vascular distribution; (b) absence of significant obstructive coronary artery disease or angiographic evidence of acute plaque rupture; (c) new electrocardiographic abnormalities (either ST-segment elevation and/or T-wave inversion) or modest elevation in cardiac troponin levels; and (d) absence of pheochromocytoma and myocarditis [8]. Complete recovery of LV dysfunction was documented in all patients within 6 months after initial presentation using transthoracic echocardiography. In addition, 20 of the 125 patients underwent follow-up CMR based on individual decisions by the treating physicians.

The control group consisted of 125 patients with anterior STEMI who participated in the CMR substudy of the “Abciximab Intracoronary versus intravenous Drug Application in STEMI (AIDA STEMI)” trial [12, 13]. The CMR substudy was conducted at 8 sites in Germany with proven experience in performing CMR examinations and included 795 patients [13]. STEMI diagnosis required the presence of ST-segment elevation ≥0.1 mV in ≥2 limb leads or ≥0.2 mV in ≥2 precordial leads in the electrocardiogram. Only patients with anterior infarction (defined as culprit lesion in the left anterior descending coronary artery) were considered eligible. The control population was chosen randomly from a list of eligible patients by personnel otherwise not involved in the study.

The study was approved by the local ethics committee and complied with the principles of the Helsinki Declaration. All patients provided written informed consent.

### CMR

**Fig. 1:**
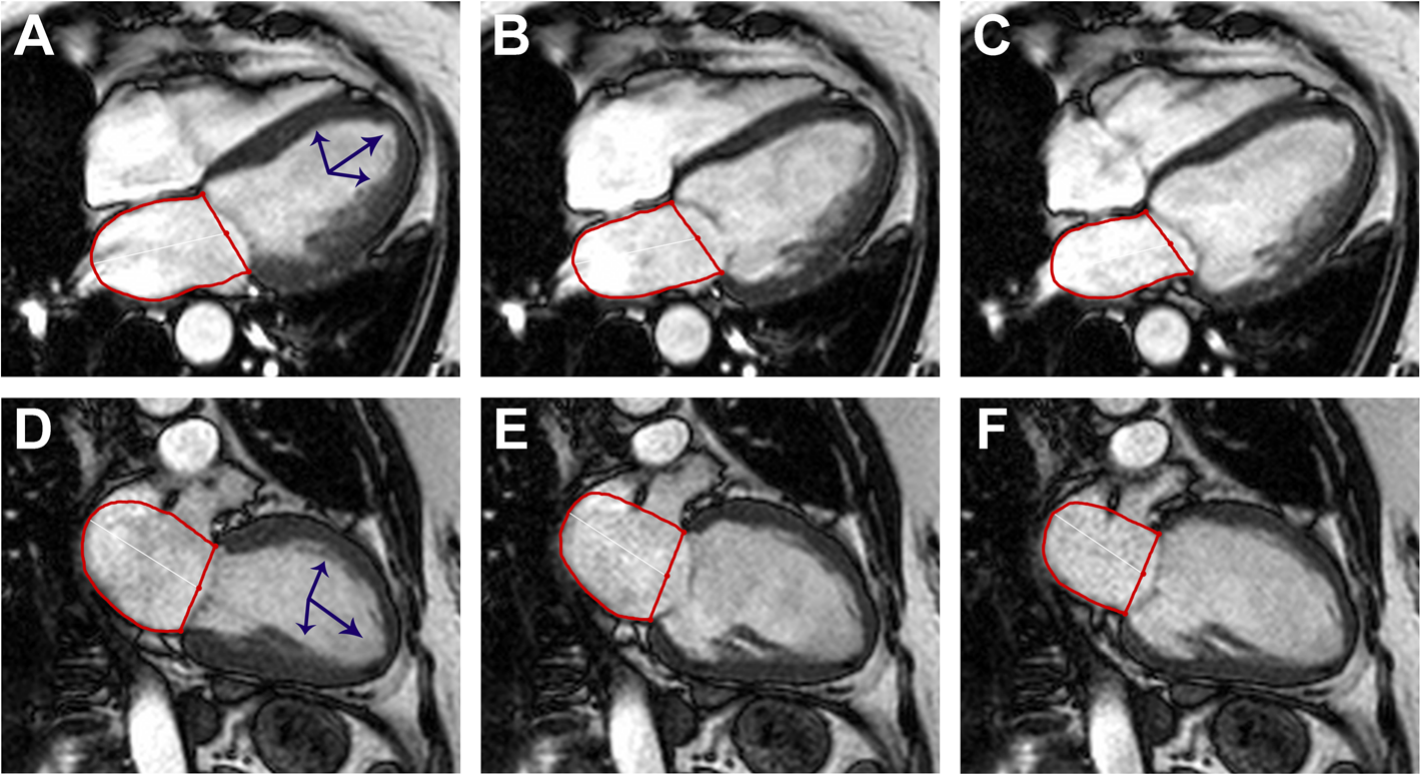
Measurement of left atrial volumes. LA area was tracked in 4-chamber (Panels **a**-**c**) and 2-chamber views (Panels **d**-**f**) for biplane assessment of LA volumes at ventricular end-systole (Panels **a** and **d**), immediately before atrial contraction (Panel **b** and **e**) as well as at ventricular end-diastole (Panels **c** and **f**). The example shows a TTS patient with typical apical ballooning (*blue arrows*)

CMR was performed within 10 days after the initial event on a 1.5- or 3.0-T magnetic resonance scanner in all patients. The standard imaging protocols for patients with myocardial infarction and suspected TTS have been used in several trials and included ECG-gated balanced steady state–free precession sequences in long-axis 2- and 4-chamber views [7, 13–15]. The imaging protocols and sequence parameters were identical in all patients. A post-hoc analysis was performed offline with certified CMR evaluation software (cmr42, Circle Cardiovascular Imaging Inc, Calgary, Alberta, Canada). LA function was assessed by using volumetric indexes derived from fractional volume changes as described previously [2–4, 10]. LA area was tracked manually in 2- and 4-chamber views excluding LA appendage and pulmonary veins to calculate LA volumes according to the biplane area-length method [16]. LA volumes were determined at ventricular end-systole (maximal LA volume, LA_max_), at ventricular diastole immediately before atrial contraction (LA_pre-A_), and at late ventricular diastole after atrial contraction (minimal LA volume, LA_min_) (Fig. 1). Total emptying fraction (EF, corresponding to global LA function and reservoir function), passive EF (corresponding to LA conduit function), and active EF (corresponding to LA booster pump function) were calculated according to the following equations [1, 10]:

$$ \mathrm{Total}\ \mathrm{E}\mathrm{F}=\frac{\left({\mathrm{LA}}_{\max }-{\mathrm{LA}}_{\min}\right)\times 100}{{\mathrm{LA}}_{\max }} $$

$$ \mathrm{Passive}\ \mathrm{E}\mathrm{F}=\frac{\left({\mathrm{LA}}_{\max }-{\mathrm{LA}}_{\mathrm{pre}-\mathrm{A}}\right)\times 100}{{\mathrm{LA}}_{\max }} $$

$$ \mathrm{Active}\ \mathrm{E}\mathrm{F}=\frac{\left({\mathrm{LA}}_{\mathrm{pre}-\mathrm{A}}-{\mathrm{LA}}_{\min}\right)\times 100}{{\mathrm{LA}}_{\mathrm{pre}-\mathrm{A}}} $$

All analyses were performed by blinded, experienced investigators. The core laboratory has proven low inter- and intraobserver variability and excellent reproducibility [17].

### Statistical analysis

Categorical variables are presented as frequencies and percentages and were compared with the Chi square test. Continuous data are reported as mean ± standard deviation in case of normal distribution or as median with interquartile range (IQR) for non-normally distributed variables. Testing for differences was performed with the Student’s t test or the Mann-Whitney U test, respectively. The Shapiro-Wilk test was used to test for normal distribution.

Among patients with TTS, LA function was compared between patients with typical apical ballooning and patients with atypical ballooning patterns (defined as midventricular or basal ballooning) as well as between patients with ongoing LV dysfunction and those with already recovered contraction (defined as LV ejection fraction >55%). Correlation between LV and LA function was analyzed with the Pearson method. The Wilcoxon signed-rank test was used to compare the total LA-EF within the cohort of patients with follow-up CMR data.

Statistical analyses were performed with SPSS (version 17.0; SPSS Inc.; Chicago, IL). A two-sided probability ≤0.05 was considered statistically significant.

## Results

A total of 250 patients were included in the present study, 125 patients with TTS and 125 patients with anterior STEMI. Of these, CMR data to evaluate LA function were available in 249 patients. One TTS patient was excluded from the analysis because of insufficient image quality. Active and passive EF could not be determined in 18 patients due to atrial fibrillation (*n* = 8 in the TTS group, *n* = 10 in the STEMI group). The time from initial presentation to CMR imaging was 2 days (IQR 2–3) in the TTS cohort and 3 days (IQR 2–3.5) in the STEMI population (*p* = 0.02).

### Baseline characteristics

The baseline clinical characteristics are summarized in Table 1. Patients with TTS were significantly older (*p* < 0.01), more frequently female (*p* < 0.01), and had a higher prevalence of hypertension (*p* = 0.02). Smoking was more prevalent in STEMI patients (*p* < 0.01).

**Table 1:** Baseline characteristics

Variable	TTS (*n* = 125)	Anterior STEMI (*n* = 125)	*p*
Age (years)	72 (61–78)	62 (51–72)	**<0.01**
Female sex	115/125 (92)	36/125 (29)	**<0.01**
Cardiovascular risk factors			
Hypertension	104/125 (83)	88/125 (70)	**0.02**
Diabetes mellitus	31/125 (25)	28/125 (22)	0.66
Hypercholesterolemia	26/125 (21)	40/125 (32)	0.06
Current smoking	20/125 (16)	46/109 (42)	**<0.01**
Killip-class 3 or 4 on admission	6/125 (5)	4/125 (3)	0.52
Stressful event	84/125 (67)	-	
Emotional	32/125 (26)	-	
Physical	52/125 (42)	-	
Ballooning pattern			
Apical	83/125 (66)	-	
Midventricular	40/125 (32)	-	
Basal	2/125 (2)	-	
Number of diseased vessels			
1	-	75/125 (60)	
2	-	37/125 (30)	
3	-	13/125 (10)	
TIMI flow grade 0 or 1 before PCI	-	77/125 (62)	
TIMI flow grade 0 or 1 after PCI	-	5/125 (4)	
Door-to-balloon time (minutes)	-	27 (21–33)	
Pain-to-balloon time (minutes)	-	220 (130–350)	
Initial LV ejection fraction (%)	47 (40–52)	50 (40–55)	0.18
Follow-up LV ejection fraction (%)^a^	60 (57–66)	-	

Values are n/N (%) or median (IQR)

*LV* left ventricular, *PCI* percutaneous coronary intervention, *STEMI* ST-segment elevation myocardial infarction, *TIMI* Thrombolysis In Myocardial Infarction, *TTS* Takotsubo syndrome

^a^Assessed with transthoracic echocardiography

*P*-values in bold type indicate a significant difference between groups

### LA size and function in TTS versus anterior STEMI

The results of CMR are illustrated in Table 2. While LA size was fairly similar in both groups considerable differences were determined regarding LA function. Patients with TTS demonstrated a significantly lower total LA-EF [44% (IQR 34–53%) versus 51% (IQR 42–56%); *p* < 0.01], passive LA-EF [21% (IQR 14–30%) versus 24% (IQR 20–29%); *p* = 0.03], and active LA-EF [29% (IQR 20–38%) versus 35% (28–42%); *p* < 0.01] compared to anterior STEMI patients.

**Table 2:** Left atrial size and function

Variable	TTS (*n* = 124)	Anterior STEMI (*n* = 125)	*p*
LA area 2-chamber view (cm^2^)			
Ventricular end-systole	21 (18–24)	22 (19–25)	**0.03**
Ventricular end-diastole	14 (12–19)	14 (12–18)	0.78
LA area 4-chamber view (cm^2^)			
Ventricular end-systole	23 (20–28)	23 (20–27)	0.90
Ventricular end-diastole	16 (12–20)	15 (12–19)	0.26
LA volume biplane (ml)			
Ventricular end-systole	72 (55–93)	74 (61–93)	0.36
Immediately before atrial contraction^a^	56 (39–70)	55 (46–68)	0.71
Ventricular end-diastole	38 (25–58)	38 (30–49)	0.67
LA function (%)			
Total EF (global function; reservoir)	44 (34–53)	51 (42–56)	**<0.01**
Passive EF (conduit)^a^	21 (14–30)	24 (20–29)	**0.03**
Active EF (booster pump)^a^	29 (20–38)	35 (28–42)	**<0.01**

Values are median (IQR)

*EF* emptying fraction, *LA* left atrial, *TTS* Takotsubo syndrome, *STEMI* ST-segment elevation myocardial infarction

^a^Patients with atrial fibrillation during CMR imaging (*n* = 8 in the TTS group and *n* = 10 in the STEMI group) were excluded from this analysis

*P*-values in bold type indicate a significant difference between groups

### LA function in TTS

**Fig. 2:**
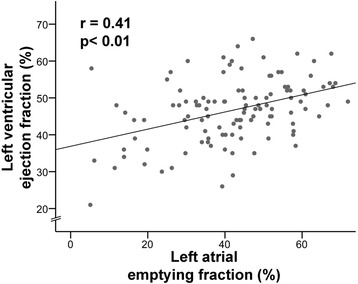
Correlation of *left atrial* and *left ventricular* function among Takotsubo syndrome patients. A significant correlation was observed between LA and LV function among patients with TTS

A detailed analysis of LA function in TTS patients did not reveal significant differences between patients with typical apical ballooning and those with atypical ballooning patterns (Table 3). However, we observed a correlation between the LV and the LA function (*r* = 0.41, *p* < 0.01; Fig. 2). Therefore, we compared LA function between TTS patients with ongoing LV dysfunction and patients with already recovered systolic function (LV ejection fraction >55%). The results exhibited a reduced total and passive LA-EF among patients with continuing LV dysfunction while the active LA-EF did not differ significantly (Table 4).

**Table 3:** Left atrial function in Takotsubo syndrome patients with typical and atypical ballooning

Variable	Typical ballooning (*n* = 82)	Atypical ballooning (*n* = 42)	*p*
Total LA-EF (global function; reservoir)	42 (33–51)	49 (36–56)	0.14
Passive LA-EF (conduit)^a^	19 (14–29)	23 (15–31)	0.30
Active LA-EF (booster pump)^a^	29 (20–38)	30 (21–38)	0.59

Values are median (IQR)

*EF* emptying fraction, *LA* left atrial

^a^Patients with atrial fibrillation during CMR imaging (*n* = 8) were excluded from this analysis, resulting in 74 patients with typical ballooning and 42 patients with an atypical ballooning pattern

**Table 4:** Left atrial function in Takotsubo syndrome patients with impaired and already recovered left ventricular function

Variable	LV ejection fraction <55% (*n* = 95)	LV ejection fraction ≥55% (*n* = 29)	*p*
Total LA-EF (global function; reservoir)	42 (30–52)	51 (42–59)	**<0.01**
Passive LA-EF (conduit)^a^	19 (14–29)	25 (20–35)	**<0.01**
Active LA-EF (booster pump)^a^	28 (16–38)	30 (24–39)	0.12

Values are median (IQR)

*EF* emptying fraction, *LA* left atrial, *LV* left ventricular

^a^Patients with atrial fibrillation during CMR imaging (*n* = 8) were excluded from this analysis, resulting in 89 patients with reduced and 27 patients with recovered LV ejection fraction

*P*-values in bold type indicate a significant difference between groups

**Fig. 3:**
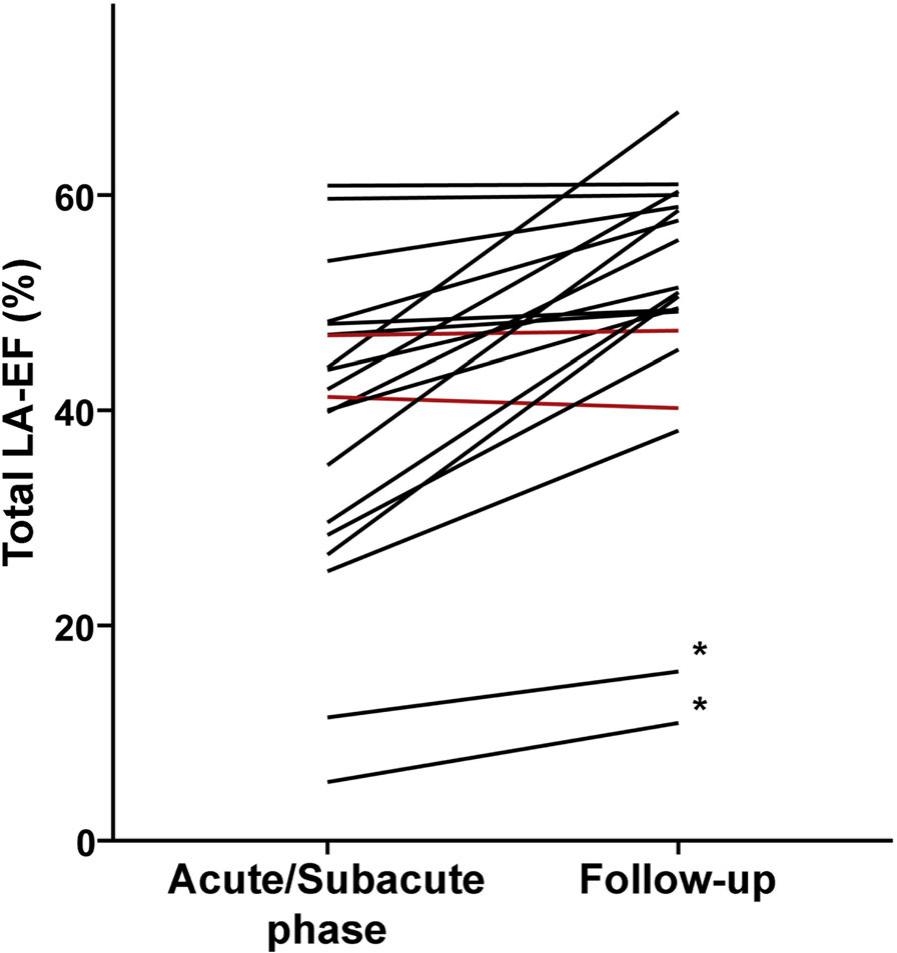
Improvement of total left atrial emptying fraction in patients with Takotsubo syndrome. Shown is the total LA-EF during the acute/subacute phase as well as at follow-up in 20 patients with TTS. Each line represents an individual patient. The figure shows a significant improvement or constantly preserved LA function in 18 patients (*black lines*) while a slight deterioration was observed in two patients (*red lines*) despite recovery of LV function (ejection fraction 53 and 68%, respectively). *Patient with permanent atrial fibrillation. LA = left atrial; EF = emptying fraction

In 20 TTS patients follow-up CMR was performed in median 3.3 months (IQR 3–5 months) after the initial event. Among these patients, the total LA-EF improved from 42% (IQR 29–48%) at the acute stage to 51% (IQR 47–60%) at follow-up (*p* < 0.01). Similarly, active LA-EF [29% (IQR 22–34%) versus 37% (IQR 32–39%); *p* < 0.01] and passive LA-EF [21% (IQR 14–27%) versus 25% (IQR 19–31%); *p* = 0.02] improved significantly. Figure 3 illustrates that 18 of 20 patients (90%) demonstrated an increased total LA-EF at follow-up while a slight drop was observed in 2 patients despite recovery of LV function.

## Discussion

The present study is the first that systematically investigated LA function and performance during the acute/subacute phase of TTS by CMR. Compared to a control group consisting of patients with anterior STEMI, TTS patients demonstrated a significantly lower total LA-EF with a deterioration of both the passive and the active phase of LA function. Furthermore, a detailed analysis of the TTS population revealed a superior LA performance in patients with already normalized LV function and a significant improvement of LA function was observed in a subgroup of patients with follow-up CMR examinations several months after the acute phase. These data indicate recovery of LA function in TTS patients similar to the LV contraction abnormalities.

### LA function in cardiovascular diseases

The left atrium serves multiple functions acting as a reservoir for pulmonary venous return and as an active contracting chamber that augments ventricular filling [1]. Thus, the left atrium significantly influences cardiovascular performance and dilates in response to either chronic volume or pressure overload. Structural and functional abnormalities of the left atrium have been reported in various cardiovascular diseases. Enlargement of the left atrium is common in patients with atrial fibrillation and might represent an important risk factor for the development of this arrhythmia [18, 19]. Moreover, the booster pump function is lost during atrial fibrillation, and the reservoir and conduit functions are also impaired [2, 20]. Elevated LV diastolic pressure in patients with heart failure leads to an increased relative contribution of active LA contraction to LV filling while the passive conduit function decreases [21]. A potential causative role of LA dysfunction in heart failure with preserved LV ejection fraction has been suggested recently [22]. Furthermore, LA size and function have been evaluated in several cohorts with ischemic or valvular heart disease as well as in patients with cardiomyopathies [1]. In addition to the available data, the present study for the first time revealed an impaired LA function in patients with TTS, which was significantly lower compared to a control population with anterior STEMI. The observed values in TTS patients are in a similar range as previously reported in patients with heart failure or chronic hypertension [4, 23, 24]. However, inter-study comparisons seem difficult considering the different methods of assessing LA parameters. Moreover, our study provides some evidence that LA performance in TTS patients recovers during follow-up similar to LV function. Whereas most patients in the sub-population with serial CMR scans demonstrated an increase in total LA-EF at follow-up, a slight decrease was observed in two patients despite normalization of LV ejection fraction. However, both patients had a long-term history of hypertension with LV hypertrophy which may also impact LA function. Moreover, delayed recovery of LV function up to 12 months after the initial event has been reported in TTS and might apply to the left atrium as well [25]. Further evidence regarding normalization of LA dysfunction in TTS is provided by a small trial in 16 patients which revealed a decreased passive emptying volume during the acute phase of TTS whereas active emptying was increased [9]. At follow-up, complete normalization of LA emptying volumes was observed.

### Reasons for LA impairment in TTS

Patients with TTS exhibit markedly reduced LV ejection fractions due to pronounced wall motion abnormalities which can be accompanied by basal hyperdynamic contraction and obstruction of the LV outflow tract. Consequently, severe LV diastolic dysfunction and substantially increased LV filling pressures have been reported in TTS patients [26]. Since LA function is closely related to LV diastolic parameters, a secondary impairment of LA performance due to an increased LV end-diastolic pressure seems reasonable. Supporting this theory, our TTS population showed a severely impaired passive LA-EF while the booster-pump function was only slightly reduced compared to previously reported data in healthy individuals [10]. Similarly, Ahtarovski et al. suggested that the high active LA contribution might have partly compensated the impaired passive LA function in their cohort [9]. However, an increased active LA-EF was not observed in our study. Apart from the theory of a secondarily impaired LA function, causative mechanisms directly affecting LA performance cannot be excluded in the absence of a sufficient pathophysiological concept for the development of TTS. Increasing evidence suggests that enhanced sympathetic activity and catecholamine excess might play a major role [27]. It is well known that neuroendocrine factors (e.g. a sustained activation of the angiotensin-aldosterone system, increases in atrial or brain natriuretic peptide) can promote LA remodeling and influence LA function although these mechanisms appear relevant in chronic diseases rather than in acute conditions as in TTS [28]. However, a direct effect of TTS on the left atrium cannot be supported by our data and remains to be proven in future trials.

### Limitations

The study population consisted of a large cohort of consecutive TTS patients. However, several patients with a severe course of the disease, particularly those with cardiogenic shock, could not undergo CMR. Therefore, the results may not be exactly applicable to these patients. Moreover, the prognostic impact of an impaired LA function in TTS patients could not be determined due to a low number of events during follow-up which does not reflect the actual outcome in TTS. Assessment of LA function was performed by using volumetric indexes only. Deformation analysis might have provided additional insights. Although CMR images were analyzed by blinded personnel, the investigators might have presumed the diagnosis in some cases based on the LV contraction pattern. Furthermore, the majority of TTS patients had a history of chronic hypertension which might have had a long-term impact on LA function. Data regarding LA parameters before the TTS event were not available. Differences regarding baseline characteristics, particularly age, sex and chronic hypertension, might have influenced the comparison between TTS and STEMI patients. Moreover, the time from presentation to CMR imaging differed between groups and might have influenced LA function. Finally, recovery of LA performance was evaluated in a small sub-population of patients with follow-up CMR data and a comparison between patients with still impaired and already recovered LV function. Therefore, the aspect of LA recovery needs further attention in future trials.

## Conclusions

In the present study, TTS was associated with a significantly lower LA-EF compared to anterior STEMI patients with a deterioration of both the passive and the active phase of LA function. However, our data indicate that LA performance recovers during follow-up similar to the LV contraction abnormalities. Consequently, transient LA dysfunction seems to be an additional feature of TTS together with the distinct impairment of LV and/or right ventricular function.

## Abbreviations

CMR: Cardiovascular magnetic resonance
EF: Emptying fraction
IQR: Interquartile range
LA: Left atrial
LV: Left ventricular
STEMI: ST-segment elevation myocardial infarction
TTS: Takotsubo syndrome
